# From Catalysis of Evolution to Evolution of Catalysis

**DOI:** 10.1021/acs.accounts.4c00196

**Published:** 2024-10-07

**Authors:** Rotem Edri, Loren Dean Williams, Moran Frenkel-Pinter

**Affiliations:** † Institute of Chemistry, The Hebrew University of Jerusalem, Jerusalem 9190401, Israel; ‡ School of Chemistry and Biochemistry, Georgia Institute of Technology, Atlanta, Georgia 30332-0400, United States; § Center for the Origins of Life, Georgia Institute of Technology, Atlanta, Georgia 30332-0400, United States; ∥ The Center for Nanoscience and Nanotechnology, The Hebrew University of Jerusalem, Jerusalem 9190401, Israel

## Abstract

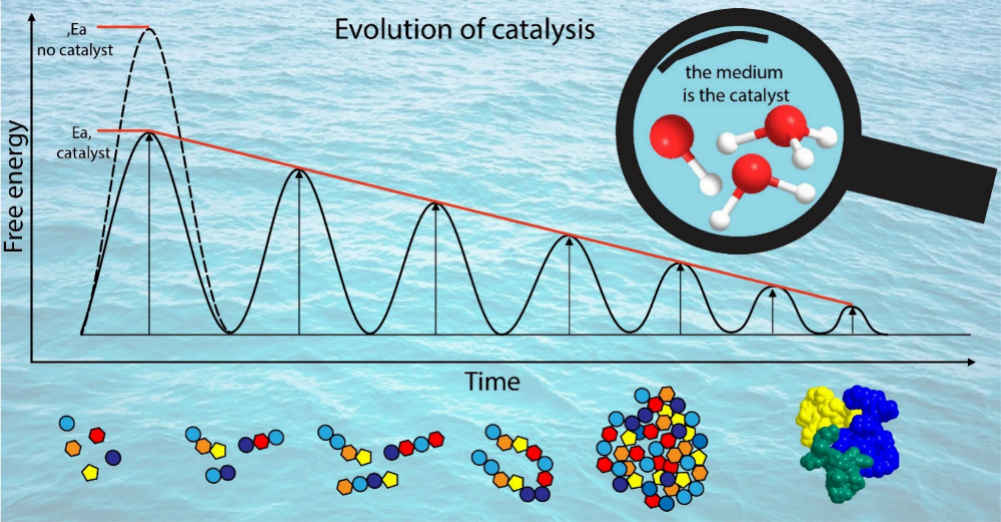

The mystery of the origins of life is one of the most difficult yet intriguing challenges to which humanity has grappled. How did biopolymers emerge in the absence of enzymes (evolved biocatalysts), and how did long-lasting chemical evolution find a path to the highly selective complex biology that we observe today? In this paper, we discuss a chemical framework that explores the very roots of catalysis, demonstrating how standard catalytic activity based on chemical and physical principles can evolve into complex machineries. We provide several examples of how prebiotic catalysis by small molecules can be exploited to facilitate polymerization, which in biology has transformed the nature of catalysis. Thus, catalysis evolved, and evolution was catalyzed, during the transformation of prebiotic chemistry to biochemistry. Traditionally, a catalyst is defined as a substance that (i) speeds up a chemical reaction by lowering activation energy through different chemical mechanisms and (ii) is not consumed during the course of the reaction. However, considering prebiotic chemistry, which involved a highly diverse chemical space (i.e., high number of potential reactants and products) and constantly changing environment that lacked highly sophisticated catalytic machinery, we stress here that a more primitive, broader definition should be considered. Here, we consider a catalyst as any chemical species that lowers activation energy. We further discuss various demonstrations of how simple prebiotic molecules such as hydroxy acids and mercaptoacids promote the formation of peptide bonds via energetically favored exchange reactions. Even though the small molecules are partially regenerated and partially retained within the resulting oligomers, these prebiotic catalysts fulfill their primary role. Catalysis by metal ions and in complex chemical mixtures is also highlighted. We underline how chemical evolution is primarily dictated by kinetics rather than thermodynamics and demonstrate a novel concept to support this notion. Moreover, we propose a new perspective on the role of water in prebiotic catalysis. The role of water as simply a “medium” obscures its importance as an active participant in the chemistry of life, specifically as a very efficient catalyst and as a participant in many chemical transformations. Here we highlight the unusual contribution of water to increasing complexification over the course of chemical evolution. We discuss possible pathways by which prebiotic catalysis promoted chemical selection and complexification. Taken together, this Account draws a connection line between prebiotic catalysis and contemporary biocatalysis and demonstrates that the fundamental elements of chemical catalysis are embedded within today’s biocatalysts. This Account illustrates how the evolution of catalysis was intertwined with chemical evolution from the very beginning.

## Key References

- Frenkel-PinterM.; HaynesJ. W.; CM.; PetrovA. S.; BurcarB. T.; KrishnamurthyR.; HudN. V.; LemanL. J.; WilliamsL. D.Selective Incorporation of Proteinaceous over Nonproteinaceous Cationic Amino Acids in Model Prebiotic Oligomerization Reactions. Proc. Natl. Acad. Sci. U. S. A.2019, 116, 16338–1634631358633 10.1073/pnas.1904849116PMC6697887.^1^ This paper describes differences in polymerization of protinogenic cataionic amino acids versus non-proteinogenic cationic amino acids as a selective force in chemical evolution.
- Frenkel-PinterM.; BouzaM.; FernándezF. M.; LemanL. J.; WilliamsL. D.; HudN. V.; Guzman-MartinezA.Thioesters Provide a Plausible Prebiotic Path to Proto-Peptides. Nat. Commun.2022, 13, 256935562173 10.1038/s41467-022-30191-0PMC9095695.^2^ This paper describes the prebiotic synthesis under various conditions of proto-peptides using mercaptoacids as robust catalysts.
- MatangeK.; RajaeiV.; Capera-AragonèsP.; CostnerJ. T.; RobertsonA.; Seoyoung KimJ.; PetrovA. S.; BowmanJ. C.; WilliamsL. D.; Frenkel PinterM.Evolution of Complex Chemical Mixtures Reveals Combinatorial Compression and Population Synchronicity. Accepted to Nat. Chem.202410.1038/s41557-025-01734-x39939341.^3^ This paper presents an experimental study of an evolved complex prebiotic system.
- Frenkel-PinterM.; RajaeiV.; GlassJ. B.; HudN. V.; WilliamsL. D.Water and Life: The Medium Is the Message. Journal of molecular evolution2021, 89, 2–1133427903 10.1007/s00239-020-09978-6PMC7884305.^4^ This paper describes the involvement of water in metabolic pathways and provides an estimation for molecular consumption and reuse of water molecules in biochemistry.

## Introduction

Prebiotic chemistry established the molecular keystones of biology, paving a path to life.^5−7^ In today’s biology, cells maintain a complex array of coordinated and simultaneous processes that are dependent on highly evolved anabolic and catabolic enzymes. Enzymes are essential keystones of life, acting as biocatalysts and regulators of cellular activity. Enzymes are made from biopolymers, such as proteins and nucleic acids. Most enzymes are proteins composed of polymerized amino acids linked via peptide bonds. Protein enzymes are responsible for replication and transcription, conducted by DNA and RNA polymerases. A few selected enzymes are based on RNA and are called ribozymes. For example, the RNA-based functional core of the ribosome catalyzes peptidyl transfer.^8^ Enzyme activities are controlled to enable synchronicity and coordination between hundreds to thousands of concerted chemical processes, which proceed within extremely short time scales (ranging from 10^–7^ to 1 s).^9^ Enzymes, such as proteases and glycosidases, increase rates of hydrolysis by orders of magnitude, thus enabling fast recycling of building blocks for the synthesis of new biopolymers.^10^ Uncatalyzed hydrolysis of biological molecules would take hundreds of thousands of years.

This astonishing array of coordinated catalytic machineries is the product of billions of years of evolution during which enzymes became increasingly complex, capable of lowering activation energies,^11−13^ regulating reaction rates, and choreographing chemical transformations across chemistry, time, and space.^14^ The complexity of enzymes is evident in their structures and functions, giving rise to high specificity, selectivity, and efficiency. Remarkably, this huge diversity of enzymes is emergent on only 20 amino acid monomers that form proteins that catalyze over 3400 different reactions (with distinct Enzyme Commission numbers) in humans.^15^ At the same time, ribozymes are emergent on only four nucleotides.^16^ The origin of such intricate, sophisticated, and precise biopolymers from prebiotic chemistry is beyond our current understanding. It is important to note that evolution is a nonlinear process;^17^ drawing direct connections between extant biology and the origins of life is impossible.

Enzymes are products of pre-Darwinian and Darwinian evolution. Stringent selection during all evolutionary phases^18−20^ appears to have enabled these advances. During early evolution, small molecules in primordial soups or on land surfaces evolved into more complex molecules.^21−32^ Among hundreds of thousands of potential prebiotic molecules only a few survived and were chosen for incorporation into contemporary biopolymers.^33−38^ It seems unlikely that most small molecules of extant biology were available in the prebiotic inventory.^17^ Instead, many are likely to be products of prebiotic or biological evolution.

**Figure 1:**
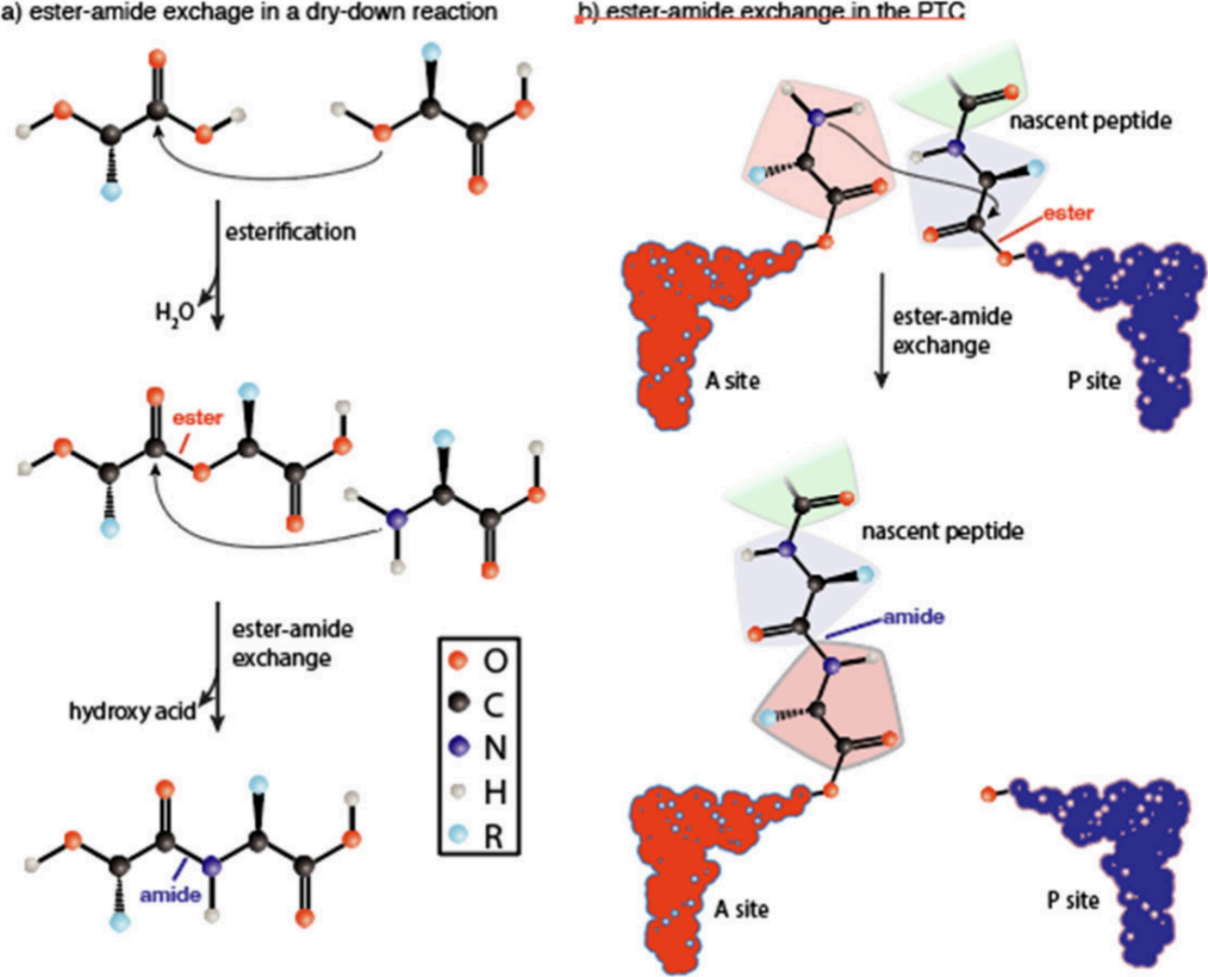
Ester–amide exchange in model prebiotic reactions and in biochemical reactions. (a) Drying amino acids with hydroxy acids makes esters that convert into peptide bonds by an attack of an amine group of an amino acid on an ester. (b) In the peptidyl transferase center of the ribosome, the amine group on an amino acid attacks an ester on the nascent polypeptide linked at the 3′ end of a tRNA, converting an ester into a peptide bond. Modified with permission from ref (47). Copyright 2020 American Chemical Society.

During chemical evolution, the diversity of the small molecules was reduced. This reduction in small molecule diversity was compensated by the increasing complexity of biopolymer sequences. Nonetheless, vestiges of the ancestral chemical processes were preserved. For example, during translation, amino acids are activated by their esterification to tRNAs. The nascent polypeptide, linked as an ester at the 3′ end of a tRNA, is transferred in the peptidyl transferase center of the ribosome to the amino group of an amino acid monomer linked as an ester at the 3′ end of another tRNA (Figure 1).^39^ The chemistry of translation resembles the chemistry of dry-down reactions of hydroxy acids and amino acids.^40−46^ In these systems, monomers link to form esters that are converted via ester–amide exchange to amides. The products are depsipeptide oligomers, which contain both amide and ester bonds (Figure 1).^1,41,45,46^ Amide bond formation is enabled through the activation of carboxylic acids during esterification reactions with hydroxy acids, as carbonyl esters serve as good electrophiles for nucleophilic attack by an amine group on the amino acid to form a peptide bond.

Recent data support models in which initial steps in the origins of life were based on chemical and physical selection. In these models, environmental conditions and an inventory of small prebiotic molecules dictated the initial course of chemical evolution. Because the Earth spins on its axis, land surfaces undergo diurnal cycling in temperature and water activity. Lower frequency seasonal cycles are superimposed on higher frequency diurnal cycles. In a dynamic and constantly changing environment, chemical systems are perpetually out of equilibrium. Some chemical species will selectively combine by condensation–dehydration reactions during a dry phase and then selectively break apart by hydrolysis during a wet phase, over and over.

In this Account, we focus on small-molecule catalysis of kinetically controlled prebiotic reactions. A catalyst, in traditional definitions, increases the rate of a reaction but is not consumed or produced over the net reaction. However, the traditional definition required reconsideration after the discovery of ribozymes. Many ribozymes catalyze phosphodiester self-scission.^48^ These ribozymes are consumed by the reaction that they catalyze and, thus, violate the traditional definition of a catalyst. Yet, they are properly considered to be catalytic. Similarly, species such as hydroxy acids catalyze the formation of peptide bonds and, in some cases but not all instances, can reside within the product (a depsipeptide). Similarly, hydroxy acids should be considered to be catalytic in the formation of peptides. Hence, it is likely that many prebiotic small molecule catalysts were altered, consumed, or produced during chemical evolution. Hence, the term “catalysis” is used here to describe chemical processes that involve intermediates that confer reduced activation energies. A catalyst is defined as a reaction participant that reduces the free energy barrier to form products by changing the reaction mechanism. This definition of a catalyst does not consider regeneration of the catalytic molecule at the end of the reaction.

Here we focus on condensation–dehydration reactions under the conditions of oscillating water activity. These systems are simultaneously kinetically controlled and near equilibrium (but never at equilibrium). Because the systems are kinetically controlled, condensation product distributions are dictated by activation energies. Because the reactions are near equilibrium, their directions alternate between formation and degradation as the water activity oscillates. A subset of building blocks is selected over the others to form oligomers. These systems are driven by a dynamic environment characterized by relentless changes in the environmental conditions. The chemical systems undergo (i) chemical selection; (ii) catalytic transformation; and (iii) increases in complexity. In this Account, we demonstrate how prebiotic catalysis could have evolved and promoted chemical evolution, leading to increasing complexity and some of the core processes of contemporary biocatalysis.

## Organic Catalysis Using Simple Prebiotic Molecules

Chemical progression and the rise of selectivity during the origins of life present some of the most challenging questions in the chemical sciences. We describe a model in which solubility, intrinsic rates of condensation, intrinsic rates of hydrolysis, catalysis, recalcitrance, and oscillating water activity are selected for some chemical species over others. One level of selection is the rate of direct oligomer formation. A second level of selection is the catalytic efficiency of oligomer formation. A third level of selection is the kinetic trapping of oligomers. Additional levels of selection were also important. Selection was progressive; production of one species enables production of a second species, etc. Direct formation of peptide bonds is prevented by high activation energies.^45,49,50^ Several approaches to overcome the high energetic cost required for peptide synthesis include mineral-mediated catalysis^51−54^ that involves the adsorption of the amino acid onto the mineral surface and the formation of the zwitterionic amino acid^51,53^ and the use of high-energy molecules such as condensing agents.^55,56^ In an alternative route that is discussed in this Account, the condensation of hydroxy acids to form ester bonds enables the production of peptide bonds. Esters catalyze amino acid condensation by lowering activation energies by about 3 kcal/mol for the formation of amide bonds through the process of ester–amide exchange.^45^ Once formed, peptide bonds are kinetically trapped. Peptide oligomers exhibit slow hydrolysis rates^49^ and offer the possibility of recalcitrant assemblies.^57^

The exploration of chemical spaces is facilitated by the differential incorporation of various monomers into oligomers. We have investigated the roots of differential oligomerization of proteinaceous and non-proteinaceous amino acids. For example, we sought to understand whether α-amino acids are favored over β-amino acids for incorporation into depsipeptides.^58^ Since both alpha amino acids and beta amino acids were prevalent on prebiotic Earth,^59^ prebiotic prevalence was probably not the reason for the selection of alpha amino acids in today’s proteins. We examined the oligomerization of both α- and β-amino acids in the presence of hydroxy acids in single-step dry-down reactions and during dry-wet cycles. Four amino acids were studied: glycine (α-amino acid), alanine (α-amino acid), β-alanine (β-amino acid), and β-aminobutyric acid (β-amino acid), as well as the analogous hydroxy acids. The results show that α-hydroxy acids more readily catalyze peptide bond formation than β-hydroxy acids. This selectivity is most likely driven by 6-membered cyclic lactone intermediates that participate in ring-opening polymerization. α-Hydroxy acids form 6-membered cyclic lactone intermediates and thus are superior catalysts for peptide bond formation compared to the corresponding β-hydroxy acids.

**Figure 2:**
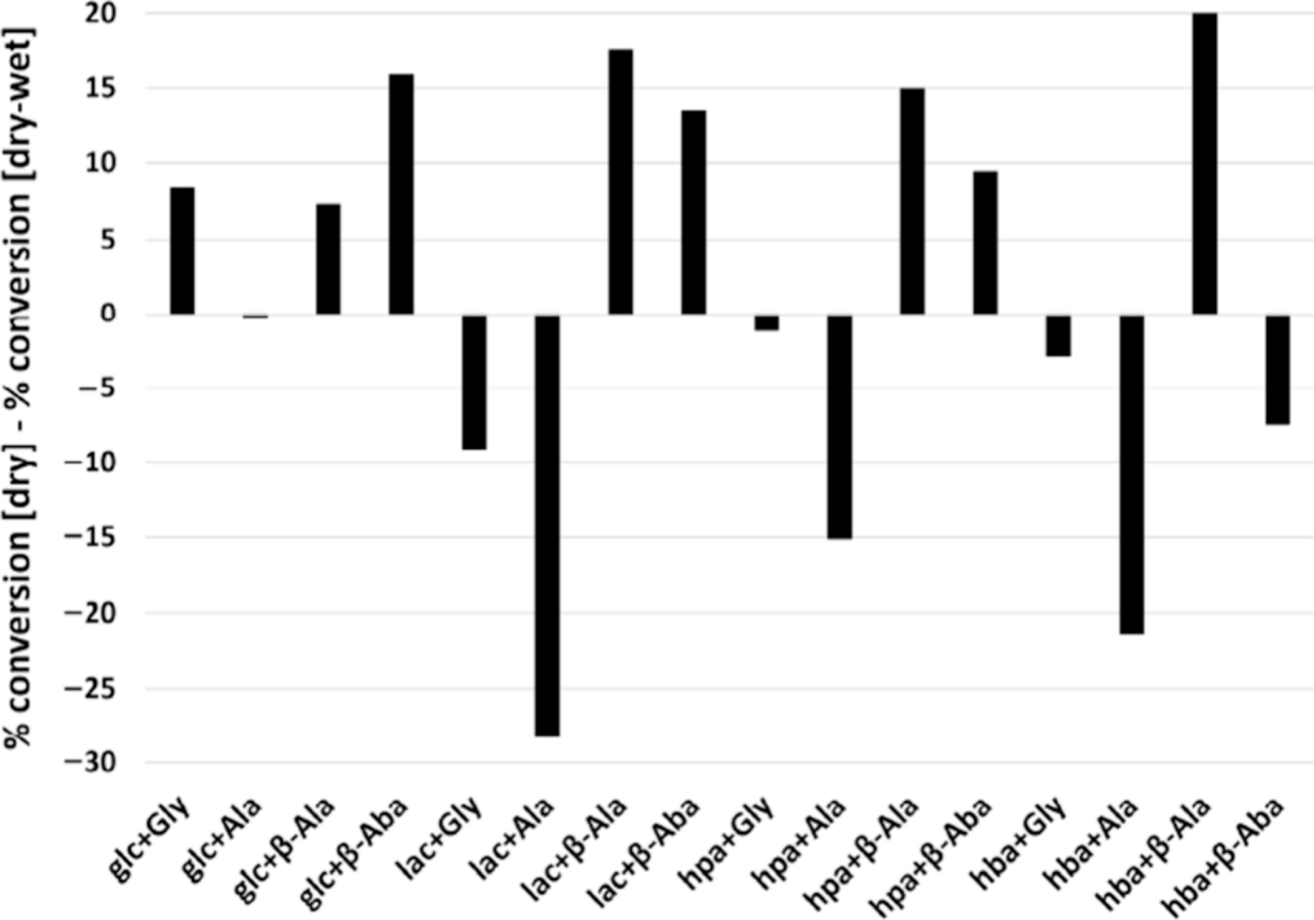
During wet-dry cycling, oligomerization of α-amino acids is favored over oligomerization of β-amino acids. Differences between amino acid conversion percentage under dry-down conditions and wet–dry cycling are illustrated. Positive values indicate greater extent of conversion under dry-down conditions, while negative values indicate greater extent of conversion under wet–dry cycling. Reproduced with permission from ref (58). Copyright 2022 MDPI, Basel, Switzerland.

The degree to which α-amino acids were incorporated into oligomers was typically lower than that of β-amino acids overall. These results are consistent with the finding that β-glutamic acid polymerizes more efficiently than its alpha analogue in the presence of 1-ethyl-3-(3-(dimethylamino)propyl)carbodiimide (EDAC).^60^ In terms of a mechanistic explanation, the nucleophilicity of the amine group of beta amino acids is expected to be greater than that of alpha amino acids due to attenuation of electron withdrawal by the carboxylic acid in beta amino acids versus alpha amino acids. Moreover, the carboxylic acids of beta amino acids, with higher p*K*_a_’s, are also better electrophiles than those of the alpha analogues. The conversion of α-amino acids to oligomers was typically greater during wet–dry cycling than during a single-step dry-down reaction. By contrast, β-amino acids were typically converted to a greater extent under single-step dry-down conditions compared to wet–dry cycling (Figure 2). Moreover, prolonged wet–dry cycling for 8 weeks resulted in gradual enrichment of α-amino acids in the resulting depsipeptides to a greater degree compared to the corresponding β-amino acids, suggesting greater evolvability of alpha over beta amino acids in depsipeptides. For instance, a mixture of alanine and lactic acid produced oligomers with up to 11-mers, from which 7-mers were alanine. The corresponding mixture of β-aminobutyric acid and lactic acid produced up to 6-mer products, 3 of which were β-aminobutyric acid.^57,61,62^ Various mechanisms and selection pressures might have affected the selection leading to alpha amino acids in biology. For example, α-amino acids were found to form dimers in the presence of trimetaphosphate while the β- and γ-amino acids did not, suggesting that oligomerization of α-amino acids is favorable via other mechanisms.^63^ Recalcitrance might also play a role in the selection, as Brack has shown that proteinaceous peptides exhibit greater resistance against hydrolysis compared to non-proteinaceous amino acids, a phenomenon that was attributed to the stable structures formed by the proteinaceous amino acids.^57,61,62^ However, comparison of recalcitrance between alpha- and beta-peptide backbones has not been demonstrated thus far.

**Figure 3:**
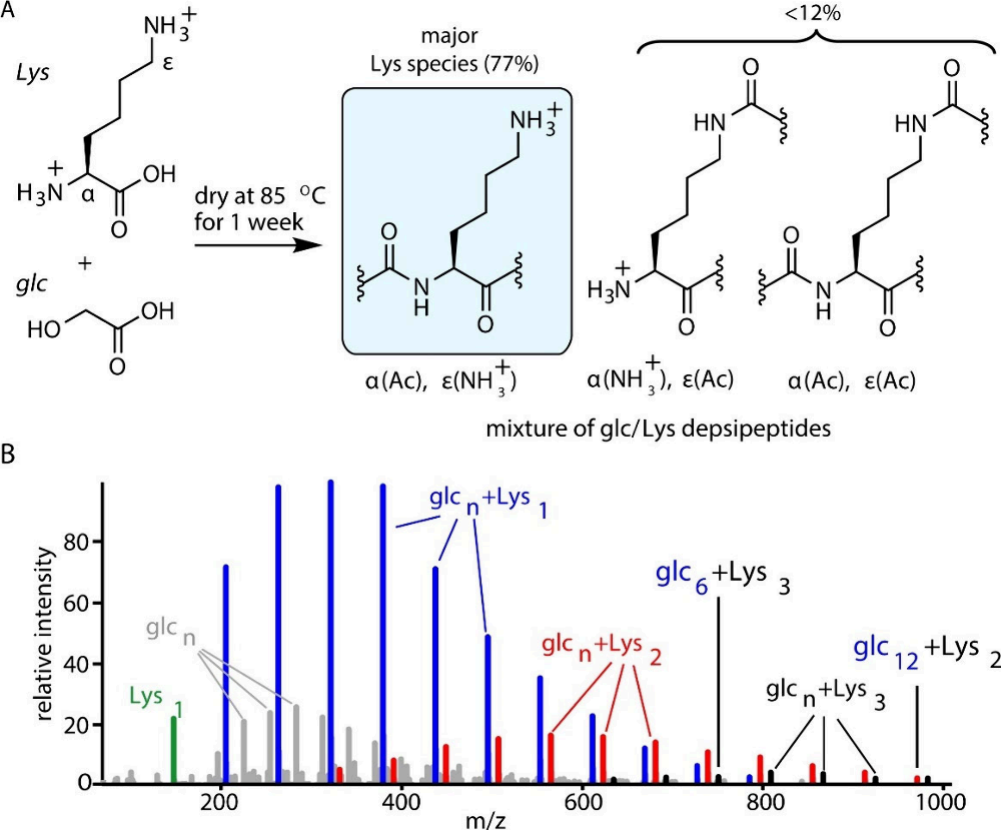
Depsipeptides containing proteinaceous cationic amino acids are formed via dry-down reactions of mixtures of hydroxy acids and cationic amino acids. (A) Examples of possible products of dry-down reactions of glycolic acid (glc) with lysine (Lys) are shown; Lys is preferentially amidated on the α-amine over the ε-amine. The percentages of products shown were determined by ^1^H-NMR analyses. (B) A mixture of glc with Lys was dried at 85 °C for 7 days, and the resulting depsipeptides were analyzed by positive-mode ESI-MS. All labeled species correspond to [M + H]^+^ ions. Reproduced with permission from ref (1). Copyright 2019 U.S. National Academy of Sciences.

Peptide bonds form preferentially between proteinogenic and non-proteinogenic cationic amino acids. Non-proteinaceous cationic amino acids such as ornithine (Orn) and 2,4-diaminobutyric acid (Dab) are thought to have been abundant on early Earth.^64−68^ Proteinaceous cationic amino acids are not considered prebiotic, even though some evidence of the possibility of abiotic synthesis or delivery has been established.^25,69−71^ We studied the propensity of several proteinaceous and non-proteinaceous cationic amino acids to undergo copolymerization with hydroxy acids into cationic depsipeptides via dry-down reaction.^1^ The presence of an amine group on the side chain of some of the investigated amino acids allows the condensation–dehydration of the amino acids at two amine group positions, resulting in two possible bonds: a canonical bond at the alpha-position and a noncanonical bond at the side chain. In a simple dry-down reaction of glycolic acid with either of the explored amino acids, we found that the proteinaceous amino acids, lysine (l-Lys), histidine (l-His), and arginine (l-Arg), react to a greater extent to form depsipeptides compared to the tested non-proteinaceous cationic amino acids. Furthermore, Lys reacted in a regioselective manner to produce alpha-amide products with not more than 12% amidation of the epsilon side-chain amine (Figure 3). Yet, the non-proteinaceous amino acids exhibited lower yields and no regioselectivity toward alpha-amidation. Moreover, the non-proteinaceous amino acids Orn and Dab underwent competing cyclization to produce lactam products.

Proteinogenic cationic amino acids also link preferentially over non-proteinogenic amino acids under competitive conditions with both classes in a common reaction vessel. For instance, in a reaction containing both l-Lys and l-Dab, Lys was incorporated preferentially over Dab into the depsipeptide oligomers. Remarkably, the presence of Lys resulted in an increase in the overall conversion of Dab compared to the corresponding binary mixture, while the presence of Dab resulted in a slightly reduced consumption of Lys but with greater regioselectivity toward alpha-amidation. These results indicate that even in a more realistic scenario in which competitive reactions occur, proteinogenic cationic amino acids are chemically preferred in peptide bond formation.

**Figure 4:**
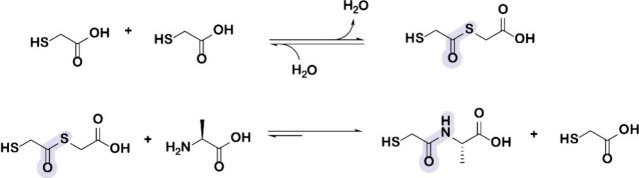
Proposed acyl substitutions during peptide bond formation (amidation) through thioester–amide exchange. Under dry conditions, mercaptoacids condense to form thioesters, which are converted to amide bonds in the presence of amino acids. Reproduced with permission from ref (2). Copyright 2022 Springer Nature.

Thiols, by forming thioesters upon reactions with carboxylic acids, can catalyze the formation of peptide bonds during wet–dry cycling. De Duve suggested a general role for thiols and thioesters in the origins of life, based on prebiotic availability and abundance in contemporary metabolism.^72^ It has been proposed that thiols were available on the early Earth when it was reductive due to impacts of iron-rich asteroids that transiently reduced the entire atmosphere.^73^ Sulfurous compounds such as hydrogen sulfide and disulfur may have been produced by volcanoes.^74^ In biology, thioesters enable the anabolism and catabolism of peptides, fatty acids, sterols, and porphyrins. Thiols and thioesters are directly involved in catalysis. Thiol proteases catalyze peptide bond hydrolysis by forming thioester low energy intermediates.^75^ We recently demonstrated that simple mercaptoacids can catalyze the reverse reaction, condensation–dehydration of amino acids, through nearly identical low energy thioester intermediates (Figure 4). The mercaptoacid route to peptide bonds offers significant advantages over the hydroxyacid route because mercaptoacids are reactive over a wider range of temperatures and pH conditions than hydroxy acids.

Formation of peptide bonds can be catalyzed by mercaptoacids that undergo thioesterification, followed by thioester amide exchange. Amide bond formation is enabled through activation of carboxylic acids during thioesterification reactions with mercaptoacids, as carbonyl thioesters serve as good electrophiles for nucleophilic attack by an amine group on the amino acid to form a peptide bond. During the exchange reaction, the hydroxy acid or mercaptoacid catalysts are released. Both hydroxy acids and mercaptoacids share a bifunctional core structure with a single difference of hydroxyl group in hydroxy acids compared to the thiol group in mercaptoacids. The replacement of the oxygen atom by the less electronegative sulfur in mercaptoacid results in a better nucleophile due to the greater polarizability of the sulfur atom. In terms of catalysis mechanism, both hydroxy acids and mercaproacids facilitate peptide bond formation by sequential steps by which either ester or thioester bonds are formed and further transformed into peptide bond by ester–amide exchange or thioester–amide exchange reactions (Figure 4).

We explored reactions involving the mercaptoacid thioglycolic acid (tg) and the amino acid alanine (Ala).^2^ These mixtures produced amide products, as verified by FTIR and NMR. HPLC and NMR demonstrated that 83% of Ala was incorporated into oligomers after a week-long single-step dry-down at 65 °C. Thioester dimers of tg formed at initial stages and were consumed at later stages to produce peptide oligomers such as tgAlaAla. The reaction mechanism involves ring-opening polymerization of a cyclic intermediate thiazinedione. The robustness of the reaction was assessed at various temperatures and pH levels. Products were observed at all tested pHs, with Ala conversion of 42% (at pH 7.0), 71% (6.5), 89% (5.5), and 90% (3.5). Some products were observed in high water activity solution, albeit at lower levels than in dried reactions.

In the path to peptide bonds, mercaptoacids such as tg appear to be more efficient catalysts than hydroxy acids. Mercaptoacids are robust catalysts across a wider range of conditions and at lower temperatures. Thioesters are more reactive toward nucleophiles such as amines, compared to oxoesters analogues, due to the loss of delocalization energy as a result of poor S–C π overlap.^76^ This difference translates into relatively low activation energies for thioester–amide exchange at lower proton concentration, lower temperatures, and higher water-activity. In summary, fine selection of peptide bond formation is enabled through simple catalytic routes using prebiotically plausible small organic molecules under a variety of environmental conditions.

## Synergy and Cooperation between Organic and Inorganic Compounds

Metal ions play significant roles in extant biology, mediating the activities of various proteins, including nitrogenases and hydrogenases,^77−80^ stabilizing folded RNA,^81^ and contributing to ribozyme catalysis.^82,83^ The catalytic role of metals and organometallic complexes in extant biology points toward ancient roots and significance of metals in prebiotic chemistry.^84^ Notably, it appears that most Earth-abundant metals are involved in biocatalysis,^85^ in particular 3d-transition metals such as iron, zinc, nickel, copper, and manganese. From a kinetic perspective, 3d-transition metals form labile complexes with their ligands, allowing rapid association–dissociation and ligand exchange,^86^ which can be essential for catalytic activity.

**Figure 5:**
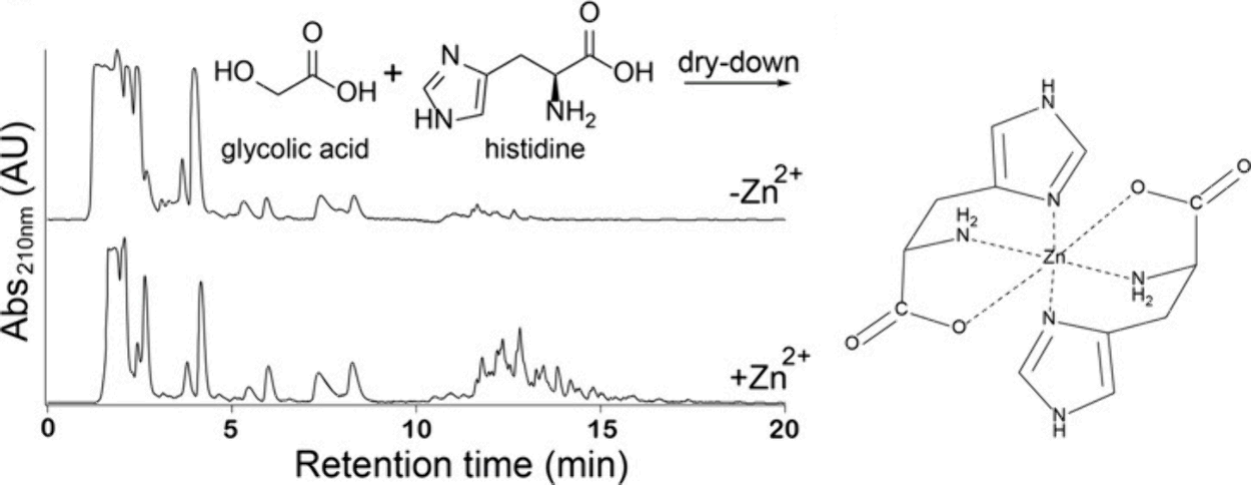
Zinc increases the yield of long His-containing depsipeptides in dry-down reactions. Histidine (His)monomer was dried with glycolic acid (glc) at a 1:1 molar ratio at 85 °C for 7 days in the presence or absence of Zn^2+^ at a 1:1 molar ratio (His:Zn^2+^). Analysis of samples via C18-HPLC showed a dramatic increase in the yield of longer oligomers in the presence of Zn^2+^. A possible coordination complex between Zn^2+^ and two His monomers is also shown. Reproduced with permission from ref (97). Copyright 2021 The Royal Society of Chemistry.

It is likely that transition metals participated in prebiotic chemistry by promoting homogeneous or heterogeneous catalysis. For instance, metal ions could have affected the synthesis of primordial peptides.^87−93^ We sought to determine the effects of transition metals on depsipeptide formation from mixtures of hydroxy acids and amino acids. We focused on histidine (His) and glycolic acid (glc) as model amino and hydroxy acids. His is involved in several metalloenzymatic elements such as the copper–histidine brace^94,95^ or zinc finger proteins.^96^ Thus, its role in enzymatic activity may be a product of prebiotic evolution. In dry-down reactions of His and glc, Zn^2+^ caused an increase in incorporation of His into long depsipeptides, but at overall lower conversion (Figure 5). In the absence of Zn^2+^, 42% of His monomers were converted into products while only 22% were converted in the presence of Zn^2+^. This effect was prominent at Zn^2+^:His 1:1 molar ratio, while at greater Zn^2+^:His ratios, oligomerization was inhibited, suggesting nonproductive association of Zn^2+^ with His. Zn^2+^ did not affect the depsipeptide formation of other amino acids, suggesting high ligand specificity. The effects of other metal ions on oligomerization of His and glc under similar dry-down conditions were also investigated. The transition metals Cu^2+^ and Co^2+^ had effects similar to those of Zn^2+^ on His incorporation into depsipeptides. Other metal ions either had no effect, as in the case of Na^+^, K^+^, and Mg^2+^, or reduced the production of His-containing depsipeptides (e.g., Ca^2+^). The association of His with transition metal ions was confirmed by circular dichroism measurements, indicating a sharp transition of His spectra during the addition of Zn^2+^, Cu^2+^, or Co^2+^. The association was attributed to the association of the imidazole moiety of His with these metal ions (Figure 5). Indeed, the hydroxy acid analogue of His exhibited similar oligomerization trends in the presence of Zn^2+^.^97^

As with other elements of today’s biology, cooperative interactions of metal ions and organic compounds may have initiated before the emergence of life. Contributions of metal ions and minerals have been proposed to protopeptide and proto-RNA^98^ synthesis and in the emergence of prebiotic catalysts in general.^99^

## Kinetically Driven Selection through Combinatorial Compression

The study of the origins of life is challenging due to sparse information, model-dependence, high complexity, and analytical challenges. We are faced with uncertainties in molecular inventories, environmental conditions, reaction pathways, selective mechanisms, and the general nature of prebiological chemical evolution. It is thought that the prebiotic milieu was rich in molecules that reacted and linked to each other in various ways.

To follow evolutionary trends in complex mixtures during wet–dry cycling, we investigated changes over time (and cycles) of a mixture containing 9 components (referred as ‘MFP Set 3’).^3^ The mixture was subjected to either single-step dry-down for 72 h or 15 iterative dry-wet cycles at 45 °C (each cycle was 48 h, one month total) under anaerobic conditions. Analysis of reaction products was monitored by HPLC, NMR, and LC-MS. We focused on global systematic trends and calculated the rate of chemical change, R_c_, as the average concentration differences between consecutive dry-wet cycles. The rate of chemical change was high at the beginning of the wet–dry cycling experiment, gradually declined by the fifth cycle, and stabilized at a nonzero value for the duration of the cycling. The data are consistent with a model in which the system continuously evolved and did not converge, or reach a steady state, throughout the course of the experiment.

Complex chemical mixtures undergoing chemical transformations tend to combinatorically explode, when a large number of ways that reactants can combine leads to large numbers of different chemical products.^100,101^ To our surprise, we observed relatively few product species after 15 cycles. The system did not “explode”, and the number of products was significantly lower than the theoretical number of product species. We used the phrase “combinatorial compression” to describe a phenomenon in which few select product species are generated from numerous diverse reactants. To investigate the phenomena of combinatorial compression, we studied how the number of reactants affected the number of products. A variety of initial mixtures with 2-components, 3-components, 4-components, 5-components, 6-components, 9-components, or 25-components were nested in such a way that subset mixtures omit reactants from parent reaction mixtures but exclude reactants not found in the parent mixture. Each 2-, 3-, 4-, 5-, or 6-component mixture was a subset of the 9-component mixture (MFP Set 3), which is a subset of a 25-component mixture. Contrary to expectations, we found that the identity of products but not the number of products changed as the number of reactants increased or the identity of the reactants changed. Specifically, upon reinitiation of a reaction with the addition of new reactants, new products appear while others disappear. We call this disappearance product subtraction. For example, the 9-component initial mixture exposed to 15 dry-wet cycles at 45 °C gave 30 products while the 25-component mixture gave 34 products. Only 20 product species were common between the two initial mixtures. Ten products were subtracted by increasing the number of reactants from 9 to 25.

We found that the balance between combinatorial explosion and compression is governed by subtle changes in the temperature. At low temperatures, combinatorial compression dominates, while at high temperatures, combinatorial explosion is observed. For varying subsets of components, as the temperature exceeds 45 °C, the number of products dramatically increases in correlation with the initial number of components. By contrast, at temperatures of 45 °C or lower, the number of products remains restricted and is hardly affected by the initial number of reactants.

Combinatorial compression appears to be kinetically controlled and is dominated by the presence of species that we term “compressors”. The basis of combinatorial compression is discussed in a subsequent theoretical investigation.^102^ Compressor molecules are relatively reactive species that can be depleted by numerous pathways in a connected system. These molecules are reactants in chemical reactions that have either significantly lower activation energy or lower Gibbs free energy than other available reactions. In principle, the phenomenon of combinatorial compression can be either kinetically or thermodynamically dominated, enabling the selection of certain products over others.^102^

The compression of the chemical space appears to be related to compression in reaction trajectories. We defined population as the number of molecules of a chemical species, trajectory as population change over wet–dry cycles, and synchronicity as similarity of trajectories of multiple species. We used a clustering algorithm to partition the trajectories into well-defined synchronous groups. The populations of the intermediate and product molecules are coordinated.

Our work provides a possible framework for understanding chemical evolution. In our model, selection is dictated in part by kinetics: select reactants form intermediates, overcoming low activation energies. During cycling, these intermediates are consumed and are either hydrolyzed or chemically transformed into products with greater durability and lower reactivity. For example, reactive species form esters or thioesters, which undergo ester–amide exchange or thioester–amide exchange to produce depsipeptides, thiodepsipeptides, or peptides. Over cycles, ester and thioester bonds disappear and amide bonds accumulate in the form of peptide-rich oligomers.

## Water: The Glue of Chemical Evolution and Catalysis

Biology, from molecules to ecosystems, is defined by water. Estuaries and rain forests are among the most productive ecosystems on Earth. Cells are around 65% by volume or 70% by weight water.^103^ Water is a requisite of life as we know it.^104^ Water is implicated in every process crucial to life, including in metabolism as reactants, intermediates, and products, and as the medium, actively fostering folding and assembly of biopolymers.^105−109^ It is impossible to think about biology without water.

Water is a powerful solvent for ions and polar substances and is a poor solvent for nonpolar substances.^110^ Water causes amphipathic molecules (with both polar and nonpolar functionalities) to form elaborate structures and proteins to fold. Water provides environmental support allowing the “activation” of enzymes by assembly.

Water shields charged species from each other.^111^ Electrostatic interactions between ions are highly attenuated in water. The electrostatic force between two ions in solution is inversely proportional to the dielectric constant of the solvent. The dielectric constant of water (80.0) is very large, over twice that of methanol (33.1) and over five times that of ammonia (15.5). Water solubilizes salts, because the attractive forces between cations and anions are significantly reduced by water.

Water is a biological catalyst in a formal sense. Biological building blocks are recycled in net reactions of protein synthesis and hydrolysis, RNA synthesis and hydrolysis, and ATP synthesis and hydrolysis. Water molecules are consumed and produced during recycling, decreasing the activation energies of the reactions. The reactions of water, in turn, are catalyzed by other species. Acids catalyze reactions of water by forming hydronium ions during acid catalysis, and bases catalyze reactions of water by forming hydroxide ions during base catalysis.

**Figure 6:**
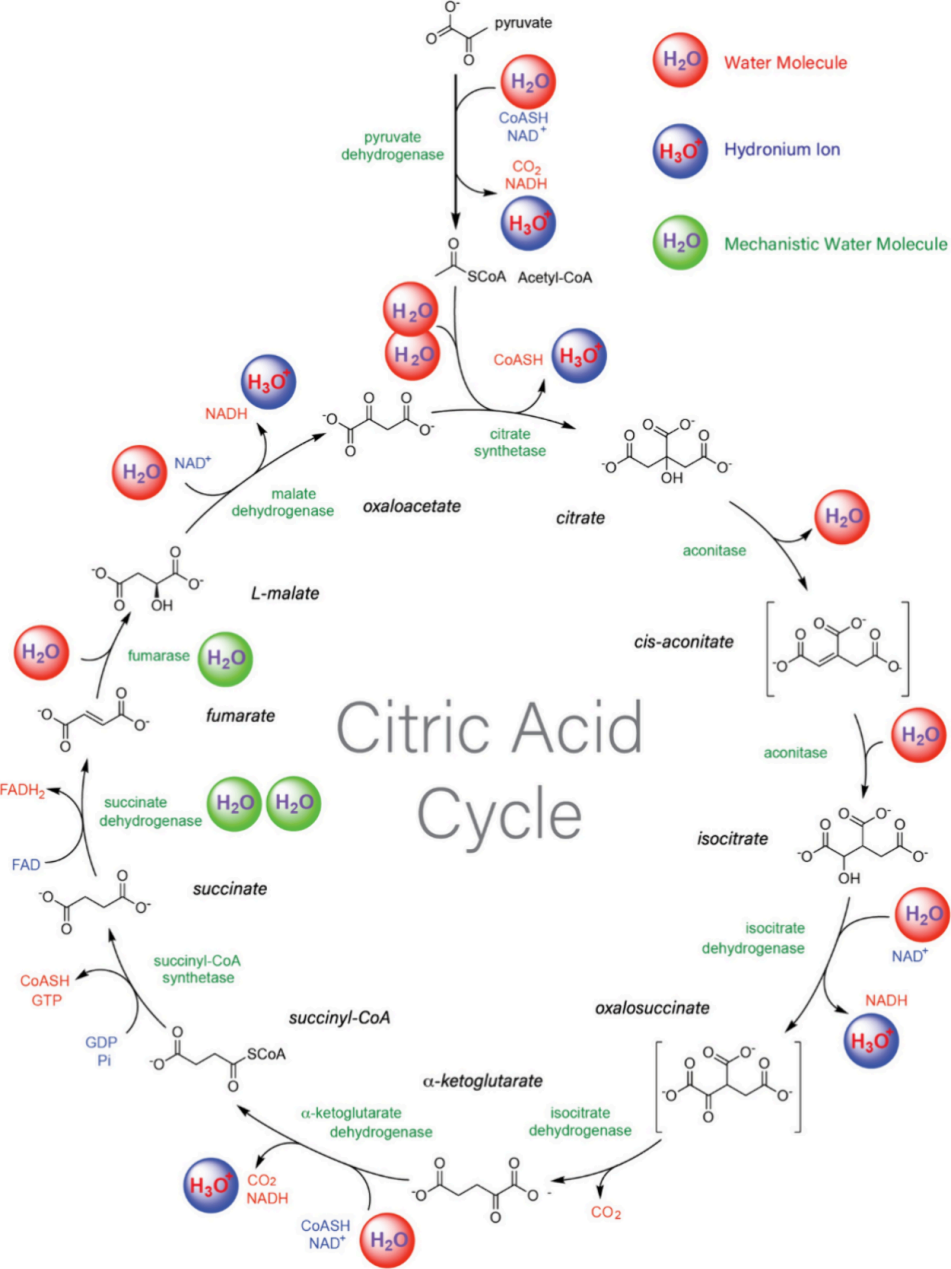
Chemical transformations of water during the Krebs cycle. In this cycle, eight enzymes (green text) catalyze a series of reactions that in total consume three water molecules, produce one water molecule, protonate three water molecules, and convert an acetyl group into two carbon dioxide molecules. Unprotonated water molecules are red spheres and protonated water molecules are blue spheres. Water molecules that are mechanistically involved in the reactions are indicated by green spheres. Reproduced with permission from ref (4). Copyright 2021 Springer Nature.

We have conducted a comprehensive survey of water chemistry in metabolism, enzymatic activity, and cell division that demonstrates the centrality of water chemistry in biology.^4^ The Krebs Cycle illustrates the significance of water chemistry (Figure 6) and other water functions. Water (i) drives folding of enzymes to functional native states; (ii) is a product in condensation–dehydration and a reactant in hydrolysis; (iii) associates with and stabilizes transition states; (iv) is a source of catalytic hydronium ions and hydroxide ions; (v) reacts with carbon dioxide to change bulk proton concentration; and (vi) coordinates metal ions^112^ and mediates metal–ion interactions with enzymes, substrates, intermediates, transition states, and products. In translation, water molecules assist ribosomal catalysis of peptidyl transfer by stabilizing the transition state via the formation of a six-member ring with a water molecule assisting proton transfer from the alpha-amine to the carbonyl oxygen.^113^

Enzymes are biocatalysts that catalyze and regulate a wide range of reactions. Our survey of the Enzyme Commission (EC) Database revealed that about one-third to half of enzymatic reactions either produce or consume water molecules.^4^ Enzymes that chemically transform water represent a plurality of enzymes in the EC database.

Water molecules are repeatedly transformed and recycled during metabolic activity. We calculated the lower limit of the frequency of chemical transformation of water during replication of *E. coli*. The calculation accounted for water transformations in protein synthesis and oxidative phosphorylation, under oxic conditions in minimal medium. Water molecules that are used mechanistically but not chemically transformed or that are transformed in other metabolic processes were omitted. The results show that approximately 88% of the water molecules in *E. coli* are chemically transformed by protein synthesis alone. Oxidative phosphorylation transforms 278% of water molecules in *E. coli*; the average water molecule is chemically recycled multiple times. Overall, the average water molecule in *E. coli* is chemically transformed at least 3.7 times during one cycle of replication. We conclude that water is the most prevalent metabolite in cells and accounts for more than 99% of all metabolites by molarity.

The diverse roles of water in extant biology suggest that it dictated the course of chemical evolution long before biology. In our model, chemical evolution required continuous chemical change, harvesting of energy from the environment, selection, increasing complexity, and self-assembly.^57^ Water was the prebiotic milieu and the reactive matrix. Building blocks were selected based on their solubility in water and ability to chemically react with water. The fittest building blocks underwent oligomerization and hydrolysis: both are directly related to water activity. Hydrolysis is an essential element of chemical evolution. Selective pressure produces chemical bonds, formed by condensation but resistant to hydrolysis. In our model, this selective pressure induces supra-molecular assemblies, which are recalcitrant (resistant to hydrolysis in the assembled state).^57^ Selection based on water chemistry in a dynamic environment leads to an increased proficiency in self-assembly and general complexity.

The environment of ancient Earth was in constant flux. Diurnal cycles were modulated by seasonal cycles that were randomly perturbed by impacts and solar flares. In our model, building blocks underwent oscillating condensation–dehydration and hydrolysis reactions. Wetting and drying on land surfaces drove oscillating condensation–dehydration reactions and hydrolysis. Oligomers that formed in the dry phase and avoided hydrolysis in the wet phase survived and persisted.^57^ The environment was always dynamic and never at equilibrium. These systems were governed by a combination of kinetic and thermodynamic effects.

Water is the conductor of evolution and the piece that holds biological constituents together. The multifunctionality of water is evident in all aspects of evolution. Water contributed to complexification as contemporary biology evolved via its multifunctionality. It seems likely that water drove and enabled chemical reactions, fostered efficient autocatalytic machineries, and conferred structure and functionality.

## Conclusions and Outlook

In this Account, we focused on the catalytic routes that may have led to the emergence of life and the emergence of contemporary biocatalysts: enzymes. Enzymes are complex machinery capable of catalyzing specific and selective chemical reactions with tremendous efficiency. Enzymes are products of long-term evolution driven by simple catalytic elements.

We suggest that environmental conditions on Early Earth provided the infrastructure for the straightforward evolution of chemical catalysis. Condensation–dehydration of reactive building blocks was accomplished in dry environments. The new, highly energetic intermediates and Earth transition metals catalyzed the formation of more resistant chemical bonds, contributing to further chemical selection. The dynamic environment of low- and high-water activity prevented the chemical systems from ever reaching equilibrium. Combined, these processes drove the complexification of the system. The keystone of chemical evolution is water, dictating the nature of chemical reactions and maintaining a dynamic chemical landscape. We stress that fundamental elements of chemical catalysis are embedded in today’s biocatalysts, illustrating how chemical evolution tells the story of the evolution of catalysis.

Our approaches to understanding the origins of life are guided by the presumption that the transition from geochemistry to biology on the ancient Earth was driven by experimentally accessible processes in environments that were not exceptional or impenetrable. The origins of life did not involve inscrutable, idiosyncratic, or one-off innovations. We assume that the transition from chemistry to biology was remarkable in sum but unremarkable during any localized step or time period. We assume that the origins of life did not require highly specific combinations of purified reagents, stringent and improbable conditions, purifications via chromatography, or teams of technically trained postdoctoral researchers.
